# Sex differences in electrolyte abnormalities indicating refeeding syndrome risk among hospitalized adolescents and young adults with eating disorders

**DOI:** 10.1186/s40337-024-01012-0

**Published:** 2024-05-24

**Authors:** Jason M. Nagata, Anthony Nguyen, Ruben Vargas, Amanda E. Downey, Anita V. Chaphekar, Kyle T. Ganson, Sara M. Buckelew, Andrea K. Garber

**Affiliations:** 1 Department of Pediatrics, University of California, San Francisco, 550 16th Street, 4th Floor, Box 0503, San Francisco, CA 94143 USA; 2 Department of Psychiatry and Behavioral Sciences, University of California, San Francisco, 550 16th Street, 4th Floor, Box 0503, San Francisco, CA 94143 USA; 3 Factor-Inwentash Faculty of Social Work, University of Toronto, 246 Bloor Street W, Toronto, ON M5S 1V4 Canada

**Keywords:** Refeeding syndrome, Electrolytes, Potassium, Magnesium, Phosphorus, Feeding and eating disorders, Male, Boys, Men, Adolescent

## Abstract

**Background:**

Refeeding syndrome is the gravest possible medical complication in malnourished patients undergoing refeeding in the hospital. We previously reported that males with malnutrition secondary to eating disorders required more calories and had longer hospital stays than females; however, sex differences in electrolyte abnormalities indicating refeeding syndrome risk remain unknown. The objective of this study was to assess differences in electrolyte abnormalities indicating refeeding syndrome risk among male and female adolescents and young adults with eating disorders hospitalized for medical instability.

**Methods:**

We retrospectively reviewed the electronic medical records of 558 patients aged 9–25 years admitted to the University of California, San Francisco Eating Disorders Program for medical instability between May 2012 and August 2020. Serum was drawn per standard of care between 5 and 7 am each morning and electrolyte abnormalities indicating refeeding syndrome risk were defined as: hypophosphatemia (< 3.0 mg/dL), hypokalemia (< 3.5 mEq/L), and hypomagnesemia (< 1.8 mg/dL). Logistic regression was used to assess factors associated with electrolyte abnormalities indicating refeeding syndrome risk.

**Results:**

Participants included 86 (15.4%) males and 472 (84.6%) females, mean (SD) age 15.5 (2.8) years. Rates of refeeding hypophosphatemia (3.5%), hypokalemia (8.1%), and hypomagnesemia (11.6%) in males hospitalized with eating disorders were low, with no statistically significant differences from females. Older age was associated with higher odds of refeeding hypophosphatemia and hypomagnesemia. Lower percent median body mass index and greater weight suppression at admission were associated with higher odds of refeeding hypophosphatemia.

**Conclusions:**

Rates of electrolyte abnormalities indicating refeeding syndrome risk were low in males hospitalized for eating disorders and rates did not significantly differ from females. Together with our finding that males have higher caloric requirements and longer hospital length of stay, the finding that electrolyte abnormalities indicating refeeding syndrome risk were not greater in males than females supports future research to evaluate the safety and efficacy of higher calorie and/or faster advancing refeeding protocols for males.

**Supplementary Information:**

The online version contains supplementary material available at 10.1186/s40337-024-01012-0.

## Abstract (pa)

Refeeding syndrome may arise as a life-threatening consequence of rapid refeeding in the setting of malnutrition. Most of the current literature and subsequent treatment protocols for managing nutritional rehabilitation and the risk of refeeding syndrome are focused on female populations. We previously showed that, compared to females, adolescent males with eating disorders need larger caloric requirements to restore medical stability. Here, we studied refeeding electrolyte abnormalities and did not find differences in male and female risk of developing refeeding electrolyte abnormalities. Older age was associated with higher odds of developing low phosphorus and low magnesium. Greater degree of malnutrition and greater weight suppression at admission were associated with higher odds of developing low phosphorus. Further studies are warranted to examine higher calorie and/or faster advancing refeeding protocols in males to assess for safety regarding development of electrolyte abnormalities indicating refeeding syndrome risk.

## Introduction

Males with eating disorders face under-recognition and undertreatment leading to medical complications, inpatient hospitalizations, and high mortality rates [1–5]. While a growing body of literature highlights the need for screening and treatment strategies among males with eating disorders, data are limited to inform sex-specific guidelines for healthcare providers [6–8]. While the impact of refeeding strategies in adolescents and young adults is an area of rigorous study, only one study to date examined sex differences in refeeding outcomes and lengths of stay [9].

During inpatient medical hospitalization, patients with malnutrition undergo refeeding treatment to restore medical stability [10]. We showed that this process requires more kilocalories (kcal) in males than females [9, 11]. However, the potential benefits of higher calorie refeeding protocols must be weighed against the risk of developing the refeeding syndrome, defined as a reduction in phosphorous, potassium, and/or magnesium, and subsequent organ dysfunction during the acute renourishment process [10, 12, 13]. Life-threatening organ dysfunction from refeeding syndrome can include cardiac failure, respiratory insufficiency, rhabdomyolysis, hemolysis, and seizures. While hypophosphatemia is viewed as a hallmark electrolyte abnormality associated with the risk of developing refeeding syndrome, hypomagnesemia appears more frequently and hypokalemia can also occur [13–17]. In a randomized control trial comparing the safety and efficacy of refeeding protocols, hypomagnesemia was the most common electrolyte shift, occurring at four times the rate of hypophosphatemia among individuals with low-calorie refeeding diets [12]. The American Society for Parenteral and Enteral Nutrition (ASPEN) recently defined the refeeding syndrome by the relative decrease in serum phosphorus, magnesium, and/or potassium, stratified as mild (10% decrease), moderate (20-30% decrease), and severe (> 30% decrease) [14]. Lack of a universally accepted definition has hampered the comparison of refeeding syndrome risk across studies and refeeding protocols [12, 15].

Prior research on refeeding syndrome has identified low weight as a predictor of refeeding syndrome in patients with anorexia nervosa (AN) [20–22]; however, these study populations were predominantly female samples and could not stratify by sex [20–22]. Studies of refeeding syndrome in males with eating disorders have been limited to small sample sizes and case studies [19, 23]. Studies are needed to fill this gap in the literature and support the development of clinical practice guidance for males with eating disorders [24]. The objective of this study was to determine the prevalence of refeeding hypophosphatemia, hypokalemia, and hypomagnesemia among a sample of male adolescents and young adults hospitalized for eating disorders and to compare sex differences in electrolyte abnormalities indicating refeeding syndrome risk. In the current study, we focus on the electrolyte abnormalities indicating refeeding syndrome risk rather than direct measures of organ dysfunction.

## Methods

### Study population

We reviewed the electronic medical records (EMRs) of 601 adolescents and young adults admitted for medical instability to an inpatient eating disorder service located at a tertiary care hospital in Northern California between May 22, 2012, and August 31, 2020. Patients ranged from age 9 to 25 at time of admission. Each patient met criteria for inpatient hospitalization based on guidelines set by the Society for Adolescent Health and Medicine, including low median body mass index (BMI) ≤ 75% for age and sex, severe bradycardia (< 50 beats/minute daytime, or < 45 beats/minute at night), hypotension (< 90/45 mmHg), hypothermia (< 96 °F, < 35.6 °C), and/or orthostasis (sustained increase in heart rate > 30 beats per minute in adults aged > 19 years, > 40 beats per minute in adolescents aged < 19 years, or sustained decreased blood pressure > 20 mmHg or > 10 mmHg diastolic) [7, 24]. Eating disorder diagnoses were made by a clinical psychologist or psychiatrist using DSM-5 criteria. Prior to May 2013, a subset of our population was diagnosed with eating disorders using DSM-IV criteria (8% of patients). These diagnoses were subsequently reclassified by the study team to an appropriate DSM-5 diagnosis upon review of clinical and psychological characteristics. All patients admitted to our medical stabilization unit meet medical admission criteria as per guidance from the Society of Adolescent Health and Medicine (e.g. vital sign instability or malnutrition criteria) [7]. Exclusion criteria included patients who did not meet DSM-5 diagnostic criteria for an eating disorder during their hospitalization (*n* = 43). The final sample included 558 patients.

### Measures

Sociodemographic data, anthropometric measurements, illness characteristics, and weight history were documented in the EMR for each participant during their hospitalization as part of standard clinical care. Serum was typically drawn in the morning between 5 and 7 am within 24 h of hospital admission and then daily, including phosphorus, potassium, and magnesium levels. Initial height and weight at admission were used to calculate BMI, kg/m^2^. Calculated BMI and median BMI for age and sex were used to calculate the patient’s percent of median BMI. Weight suppression was defined as the difference between the patient’s highest reported weight per chart review and their admission weight, divided by patient’s highest reported weight.

### Nutritional rehabilitation protocol

The Eating Disorders Program at the University of California, San Francisco follows specific protocols regarding admission criteria, laboratory evaluation, caloric prescriptions and diet escalation, food macronutrient content, physical activity allowance, and discharge criteria, in line with clinical guidance from the Society for Adolescent Health and Medicine [7]. Most patients commenced at 2,000 kcal/day and advanced by 200 kcal each day; we previously reported that average (SD) starting diet prescription was 1985 (233) kcal [9]. Electrolytes were checked daily in the morning for a minimum of seven days to evaluate for risk of developing refeeding syndrome (unless the patient was discharged in under seven days) [12]. Refeeding hypophosphatemia, hypokalemia, or hypomagnesemia were defined as phosphorus (< 3 mg/dL), potassium (< 3.5 mEq/L), or magnesium (< 1.8 mg/dL); combined refeeding risk refers to the presence of any one of these abnormalities. Refeeding syndrome risk was indicated by the incidence of electrolyte abnormalities beginning on day 2 in the hospital, after one full day of refeeding in the hospital (i.e., low potassium, magnesium, or phosphorus present at admission prior to the onset of the refeeding protocol were not included towards the classification of electrolyte abnormalities indicating refeeding syndrome risk, but the patients were still included in the study to evaluate for subsequent laboratory abnormalities). All laboratory samples were collected using a standardized protocol and analyzed at a Clinical Laboratory Improvement Amendments (CLIA)-certified laboratory, following federal standards and regulations [12]. In order to minimize electrolyte abnormalities due to purging, several protocols and precautions are implemented for any patient with a history of purging, including a patient care attendant observing the patient during all meals/snacks and for a period following the completion of the meal/snack, taping/sealing sinks in the room, and limiting showers to early mornings before meals.

### Ethics

This retrospective chart review involving human participants was approved by the Institutional Review Board (IRB) of the University of California, San Francisco (20-30323). All study components are in accordance with the ethical standards set by the 1963 Helsinki Declaration, its later amendments, and the institutional and national research committee.

### Statistical analysis

Statistical data analysis was conducted using Stata 15.1 (StataCorp LP, College Station, TX). Unadjusted sex differences in continuous variables (e.g., age, BMI) were compared using independent sample t-tests. Unadjusted sex differences in categorical variables (e.g., race/ethnicity, eating disorder diagnosis) were compared using Chi-squared or Fisher’s exact tests. We examined rates of electrolyte abnormalities indicating refeeding syndrome risk in the entire sample and in the subsample with anorexia nervosa, restricting subtype.

We first ran simple logistic regression models to determine unadjusted associations between independent variables (male sex, age, ethnicity, eating disorder diagnosis, duration of illness, percent median BMI, weight suppression at admission, prescribed kcal at admission, and prescribed kcal at discharge) with refeeding hypophosphatemia, hypokalemia, hypomagnesemia, or a combined electrolyte abnormality variable separately. Additional logistic regression models adjusted for percent median BMI, which is standardized across sex and age and previously shown to relate to the risk of developing refeeding syndrome, were conducted for each outcome, respectively.

## Results

Table 1 describes the demographic and clinical characteristics of 86 (15.4%) male and 472 (84.6%) female study participants, stratified by sex. Mean age was 15.5 years, 59.7% identified as non-Hispanic white, and 63.6% had anorexia nervosa. Mean (SD) percent median BMI was 87.5 (14.2). Admission serum phosphorus, potassium, and magnesium did not differ by sex. Incidence of electrolyte abnormalities indicating refeeding syndrome risk were 8.1% hypophosphatemia, 10.6% hypokalemia, and 19.2% hypomagnesemia, with a combined risk of 26.5%. Although rates of refeeding hypophosphatemia (M = 3.5%, F = 8.9%, *p* = 0.090), hypokalemia (M = 8.1%, F = 11.0%, *p* = 0.425), hypomagnesemia (M = 11.6%, F = 20.6%, *p* = 0.053), and combined risk (M = 19.8%, F = 27.8%, *p* = 0.123) were lower in males than in females, the differences were not statistically significant and the effect sizes for the differences were small. Findings in the subsample with anorexia nervosa, restricting subtype were similar (Appendix A).

**Table 1:** Demographic and clinical characteristics of adolescents and young adults hospitalized for restrictive eating disorders by sex

Characteristic	Total (*N* = 558)	Sex			
		Male (*n* = 86)	Female (*n* = 472)	p ^a^	Effect size^b^
Age, years, mean (SD)	15.5 ± 2.8	15.6 ± 2.6	15.5 ± 2.8	0.612	0.060
Race/ethnicity, n (%)				**0.026**	0.094
Asian or NHOPI^c^	43 (7.7)	5 (5.8)	38 (8.1)		
Hispanic	97 (17.4)	25 (29.1)	72 (15.3)		
Multiracial	29 (5.2)	4 (4.7)	25 (5.3)		
Non-Hispanic Black or African American	10 (1.8)	4 (4.7)	6 (1.3)	`	
Non-Hispanic White	333 (59.7)	42 (48.8)	291 (61.7)		
Other	28 (5.0)	3 (3.5)	25 (5.3)		
Unknown/declined	18 (3.2)	3 (3.5)	15 (3.2)		
Diagnosis, n (%)				**< 0.001**	0.152
Anorexia nervosa	355 (63.6)	40 (46.5)	315 (66.7)		
Avoidant restrictive food intake disorder (ARFID)	36 (6.45)	13 (15.1)	23 (4.9)		
Binge-eating disorder	1 (0.2)	0	1 (0.2)		
Bulimia nervosa	10 (1.8)	1 (1.2)	9 (1.9)		
Other specified feeding and eating disorder (OSFED)					
Atypical anorexia nervosa	90 (16.1)	21 (24.4)	69 (14.6)		
Purging disorder	2 (0.4)	0	2 (0.4)		
Unspecified feeding and eating disorder (UFED)	64 (11.5)	11 (12.8)	53 (11.2)		
BMI, kg/m^2^, mean (SD)	17.7 ± 3.0	18.1 ± 3.9	17.6 ± 2.7	0.100	0.193
% median BMI, mean (SD)	87.5 ± 14.2	88.6 ± 17.9	87.3 ± 13.4	0.417	0.095
Weight suppression at admission, mean % (SD)^*d*^	18.6 ± 10.3	18.4 ± 11.9	18.7 ± 10.0	0.237	0.022
Duration of illness, months, mean (SD)^*e*^	15.4 ± 19.7	14.4 ± 16.4	15.6 ± 20.3	0.621	0.061
Prescribed kcal, mean (SD)					
Admission	1984 ± 230	2016 ± 325	1978 ± 208	0.155	0.167
Discharge^*f*^	3124 ± 566	3777 ± 705	3005 ± 444	**< 0.001**	1.570
Admission serum electrolyte laboratory values, mean (SD)					
Magnesium	2.1 ± 0.2	2.1 ± 0.2	2.1 ± 0.2	0.167	0.162
Phosphorus	3.9 ± 1.9	4.0 ± 0.7	3.9 ± 0.6	0.127	0.179
Potassium	3.9 ± 0.6	3.9 ± 0.7	3.9 ± 0.5	0.244	0.137
Refeeding electrolyte laboratory values, n (%)					
Refeeding hypomagnesemia (< 1.8 mg/dL)	107 (19.2)	10 (11.6)	97 (20.6)	0.053	0.082
Refeeding hypophosphatemia (< 3.0 mg/dL)	45 (8.1)	3 (3.5)	42 (8.9)	0.090	0.072
Refeeding hypokalemia (< 3.5 mmol/L)	59 (10.6)	7 (8.1)	52 (11.0)	0.425	0.034
Combined electrolyte abnormalities indicating refeeding syndrome risk^*g*^	148 (26.5)	17 (19.8)	131 (27.8)	0.123	0.065

Boldface indicates *p* < 0.05

^*a*^ P-value is for t-tests for continuous variables or Pearson’s chi square tests (or Fisher’s exact test as appropriate) for categorical variables. For race/ethnicity, p-value is for Pearson’s chi square test comparing a binary race/ethnicity variable (non-Hispanic white vs. all other race/ethnicities) by sex (male vs. female). For diagnosis, p-value is for Pearson’s chi square test comparing a binary diagnosis variable (anorexia nervosa vs. all other diagnoses) by sex (male vs. female)

^*b*^ Cohen’s d for continuous variables, Cramer’s V for categorical variables

^*c*^ NHOPI = Native Hawaiian and Other Pacific Islanders

^*d*^ Due to missing data, the weight suppression rate has a sample size of 500 (Male *n* = 75; Female *n* = 425)

^*e*^ Due to missing data for duration of illness, a sample size of 528 was utilized (Male *n* = 78; Female *n* = 450)

^*f*^ Due to missing data for prescribed kcal at discharge, a sample size of 557 was utilized (Male *n* = 86; Female *n* = 471)

^*g*^ A variable indicating refeeding hypomagnesemia or hypophosphatemia or hypokalemia

Table 2 identifies the factors associated with refeeding hypophosphatemia, hypokalemia, hypomagnesemia, and combined risk in both unadjusted models and models adjusted for percent median BMI. There were no significant associations with male (compared to female) sex. In unadjusted logistic regression models, older age (OR 1.18, 95% CI 1.06–1.30, *p* = 0.001), greater weight suppression at admission (OR 1.04, 95% CI 1.01–1.07, *p* = 0.003), and percent median BMI (OR 0.96, 95% CI 0.94–0.99, *p* = 0.004) were associated with refeeding hypophosphatemia. In models adjusted for percent median BMI in the same model, older age (OR 1.15, 95% CI 1.03–1.27, *p* = 0.009) and greater weight suppression at admission (OR 1.04, 95% CI 1.00–1.07, *p* = 0.024) remained associated with higher odds of refeeding hypophosphatemia. Older age was associated with refeeding hypomagnesemia in both unadjusted models (OR 1.10, 95% CI 1.02–1.18, *p* = 0.012) and models adjusted for percent median BMI (OR 1.09, 95% CI 1.02–1.18, *p* = 0.018). Additionally, older age showed an association with combined refeeding syndrome risk in both unadjusted models (OR 1.09, 95% CI 1.02–1.16, *p* = 0.012) and models adjusted for percent median BMI (OR 1.08, 95% CI 1.01–1.16, *p* = 0.025). None of the investigated factors were found to be associated with refeeding hypokalemia.

**Table 2:** Factors associated with refeeding hypophosphatemia, hypokalemia, hypomagnesemia, and combined refeeding syndrome risk

	Unadjusted		Adjusted for % median BMI	
Independent variables	OR (95% CI)^a^	p	OR (95% CI)^b^	p
Refeeding hypophosphatemia (low phosphorus)				
Male sex	0.37 (0.11, 1.22)	0.103	0.37 (0.11, 1.22)	0.101
Age, years	**1.18 (1.06, 1.30)**	**0.001**	**1.15 (1.03, 1.27)**	**0.009**
White (vs. non-White)	1.39 (0.73, 2.64)	0.321	1.29 (0.67, 2.47)	0.442
Anorexia nervosa (vs. non-anorexia nervosa)	1.31 (0.68, 2.53)	0.415	0.96 (0.48, 1.90)	0.906
Duration of illness	1.01 (1.00, 1.02)	0.104	1.01 (1.00, 1.02)	0.158
Percent median BMI	**0.96 (0.94, 0.99)**	**0.004**	--	--
Weight suppression at admission^*c*^	**1.04 (1.01, 1.07)**	**0.003**	**1.04 (1.00, 1.07)**	**0.024**
Refeeding hypokalemia (low potassium)				
Male sex	0.72 (0.31, 1.63)	0.427	0.72 (0.32, 1.65)	0.437
Age, years	0.95 (0.85, 1.05)	0.285	0.94 (0.85, 1.04)	0.230
White (vs. non-White)	1.06 (0.61, 1.85)	0.824	1.04 (0.60, 1.81)	0.895
Anorexia nervosa (vs. non-anorexia nervosa)	1.49 (0.83, 2.70)	0.184	1.44 (0.77, 2.69)	0.257
Duration of illness	0.99 (0.97, 1.01)	0.332	0.99 (0.97, 1.01)	0.325
Percent median BMI	0.99 (0.97, 1.01)	0.431	--	--
Weight suppression at admission^*c*^	1.01 (0.98, 1.03)	0.540	1.01 (0.98, 1.03)	0.652
Refeeding hypomagnesemia (low magnesium)				
Male sex	0.51 (0.25, 1.02)	0.057	0.51 (0.26, 1.03)	0.060
Age, years	**1.10 (1.02, 1.18)**	**0.012**	**1.09 (1.02, 1.18)**	**0.018**
White (vs. non-White)	1.42 (0.91, 2.21)	0.118	1.39 (0.89, 2.17)	0.149
Anorexia nervosa (vs. non-anorexia nervosa)	1.31 (0.84, 2.05)	0.237	1.23 (0.76, 1.98)	0.396
Duration of illness	0.99 (0.98, 1.01)	0.327	0.99 (0.98, 1.01)	0.311
Percent median BMI	0.99 (0.98, 1.01)	0.257	--	--
Weight suppression at admission^*c*^	1.00 (0.98, 1.02)	0.966	1.00 (0.98, 1.02)	0.824
Combined electrolyte abnormalities indicating refeeding syndrome risk^*d*^				
Male sex	0.64 (0.36, 1.13)	0.125	0.65 (0.37, 1.14)	0.134
Age, years	**1.09 (1.02, 1.16)**	**0.012**	**1.08 (1.01, 1.16)**	**0.025**
White (vs. non-White)	1.30 (0.88, 1.91)	0.192	1.25 (0.84, 1.84)	0.271
Anorexia nervosa (vs. non-anorexia nervosa)	1.24 (0.83, 1.84)	0.286	1.10 (0.73, 1.68)	0.645
Duration of illness	1.00 (0.99, 1.01)	0.423	1.00 (0.98, 1.01)	0.391
Percent median BMI	0.99 (0.97, 1.00)	0.063	--	--
Weight suppression at admission^*c*^	1.01 (0.99, 1.03)	0.159	1.01 (0.99, 1.03)	0.260

Boldface indicates *p* < 0.05

^*a*^ Unadjusted represents outputs from logistic regression analyses with the listed independent variable and refeeding hypophosphatemia, hypokalemia, hypomagnesemia, or combined refeeding risk as the dependent variable

^*b*^ Adjusted represents outputs from logistic regression analyses with the listed independent variable and refeeding hypophosphatemia, hypokalemia, hypomagnesemia, or combined refeeding risk as the dependent variable, adjusted for percent median BMI.

^*c*^ Weight suppression is expressed as a percentage

^*d*^ A variable indicating hypomagnesemia (low magnesium) or hypophosphatemia (low phosphorus) or hypokalemia (low potassium)

## Discussion

In this study of 558 adolescents and young adults, we did not find significant sex differences in electrolyte abnormalities as indicators of risk for developing the refeeding syndrome. Overall, rates of refeeding hypophosphatemia (3.5%), hypokalemia (8.1%), and hypomagnesemia (11.6%) in males hospitalized with eating disorders were similar to patients undergoing higher calorie refeeding (starting 2000 kcal) in a clinical trial [12]. This was expected given that this study population received a very similar refeeding approach, starting at 2016 kcal in males and 1978 kcal in females and the same electrolyte monitoring and replacement protocol was used. Notably higher rates of electrolyte abnormalities in prior studies [25] were due to overestimation by counting patients who received prophylactic electrolyte supplementation or treatment of declining (but not low) serum levels as having had an electrolyte abnormality. Use of a standardized protocol to monitor and replace only serum levels below prespecified thresholds in the present study likely gives a more accurate representation of refeeding risk.

The incidence of electrolyte abnormalities indicative of refeeding syndrome risk is of particular interest in male patients as males have higher refeeding goal diets and longer hospital stays than females, and therefore may be at added risk of developing refeeding syndrome [9]. We did not find statistically significant differences in rates of electrolyte abnormalities indicative of refeeding syndrome. Although the rates of electrolyte abnormalities indicative of refeeding syndrome in males were qualitatively lower than in females, the effect sizes were small. A greater degree of malnutrition has traditionally been associated with higher refeeding risk based on historical studies in patients with AN [26]. Indeed, we did find that lower percent median BMI was associated with higher odds of refeeding hypophosphatemia. However, when controlling for presentation percent median BMI, we then showed that greater weight suppression was independently associated with higher odds of refeeding hypophosphatemia. These findings reinforce the importance of weight history in assessing risk and are consistent with prior studies in atypical AN showing that weight suppression predicts medical risk [27, 28].

We showed that older age was associated with higher odds of refeeding hypophosphatemia and hypomagnesemia [29]. This may be explained by the longer duration of illness in older adolescents and young adults when compared to younger adolescents. A prior study in patients with AN and atypical AN demonstrated an association between longer duration of weight loss and lower serum phosphorus at presentation [27]. However, in a study of hypophosphatemia during the course of refeeding, Whitelaw et al. (2018) did not find associations with duration or recency of weight loss [27]. Thus, although it is plausible that total body mineral stores become increasingly depleted over longer illness duration, more studies are needed to determine whether this contributes to more frequent electrolyte abnormalities during renourishment. Perhaps the most important implication of this finding is that patients with delayed or missed diagnoses will be at higher risk due to longer duration of illness. This puts males at particular risk given studies showing inadequate assessment of eating disorders in male patients and delayed referrals to specialty care.

Although males and females were prescribed similar kcals on admission, males overall required higher daily caloric requirements by discharge (3777 kcals) compared to females (3005 kcals) to establish medical stability. We previously showed that this contributed to longer hospitalizations in males [9, 30]. While this did not translate into greater electrolyte abnormalities indicating refeeding syndrome risk, extended duration of stays confers greater financial costs and increases the risk of hospital-acquired conditions for patients [31]. Thus, future research is needed to optimize refeeding outcomes in male patients, particularly among samples with greater sex parity to increase power for detecting potential sex differences. Although higher caloric refeeding strategies have been shown to restore medical stability and shorten hospital stay, these strategies were not sex-stratified [12]. Potential trials in adolescent and young adult males with eating disorders may include either a larger initial caloric diet or more aggressive caloric advances while monitoring electrolytes to optimize treatment protocols in malnutrition-associated medical instability.

### Limitations and strengths

Limitations of this retrospective study include its observational nature which limits inferences of causality. With findings conducted at a single site from a tertiary care hospital in Northern California, generalizability may be limited to other populations given differences in regional sociodemographic factors and diets. As patients were aged 9 to 25 at time of admission, findings of this study may not be applicable to older adult populations. Patients who met DSM-IV criteria also met DSM-5 criteria but were diagnosed prior to the publication of DSM-5. To elucidate sex differences, we retained individuals originally diagnosed with DSM-IV criteria to maximize power, ensuring as large of a male sample size as possible. There are limitations to the use of BMI and percent median BMI as they cannot differentiate body composition (e.g., fat mass, lean mass), which may differ by sex among adolescents with eating disorders [32]. Despite these limitations, we report the percent median BMI in this study as the Society for Adolescent Health and Medicine recommends the use of percent median BMI to classify degree of malnutrition in patients with restrictive eating disorders [7].

Strengths of this study include a robust patient sample over the course of 8 years and a relatively high male clinical sample (*n* = 86, 15%). This is the first study to our knowledge that evaluates the association of sex with refeeding hypophosphatemia, hypokalemia, or hypomagnesemia, and we adjusted regression analyses for percent median BMI. The lack of sex differences in both individual and compound variables of electrolyte abnormalities indicating refeeding syndrome risk show great promise towards tailoring current refeeding protocols towards more aggressive treatment and advancement of diets in adolescent male populations with eating disorders.

## Conclusion

Males continue to be underrepresented in eating disorder research and often receive treatment based on data derived from predominantly female samples despite their higher caloric requirements and longer hospitalizations [33]. Higher initial caloric diets and rapid advancement of diet is associated with faster restoration of medical stability and shorter hospital stays, but such studies are not sex stratified [12]. Our findings that males and females with eating disorders do not have statistically significant electrolyte abnormalities indicating risk of refeeding syndrome suggests that higher or more aggressive refeeding protocols in male populations may be warranted, though future studies are needed.

### Electronic supplementary material

Below is the link to the electronic supplementary material.

Supplementary Material 1

## Data Availability

The data that support the findings of this study are available on request from the corresponding author, J.M.N. The data are not publicly available due to confidentiality restrictions (e.g., their containing information that could compromise the privacy of research participants).

## Abbreviations

CLIA: Clinical Laboratory Improvement Amendments
EMR: Electronic medical records
BMI: Body Mass Index

## References

[1] Murray SB, Nagata JM, Griffiths S, Calzo JP, Brown TA, Mitchison D (2017). The enigma of male eating disorders: a critical review and synthesis. Clin Psychol Rev.

[2] Nagata JM, Ganson KT, Murray SB (2020). Eating disorders in adolescent boys and young men: an update. Curr Opin Pediatr.

[3] Forrest LN, Smith AR, Swanson SA (2017). Characteristics of seeking treatment among U.S. adolescents with eating disorders. Int J Eat Disord.

[4] Nagata JM, Ganson KT, Golden NH. Medical complications of eating disorders in boys and men. Eating disorders in boys and men. Cham: Springer Int Publishing; 2021.

[5] Quadflieg N, Strobel C, Naab S, Voderholzer U, Fichter MM (2019). Mortality in males treated for an eating disorder—A large prospective study. Int J Eat Disord.

[6] National Guideline Alliance (UK). Eating disorders: recognition and treatment. London: National Institute for Health and Care Excellence (NICE);2017. https://www.nice.org.uk/guidance/ng69/chapter/Recommendations-for-research. Accessed 1 Apr 2024. 28654225

[7] Medicine, Golden NH, Katzman DK, Sawyer SM, Ornstein RM, Rome ES, Society for Adolescent Health and (2015). Position paper of the Society for Adolescent Health and Medicine: medical management of restrictive eating disorders in adolescents and young adults. J Adolesc Health.

[8] The American psychiatric association practice guideline for the treatment of patients with eating disorders. https://psychiatryonline.org/doi/epdf/10.1176/appi.books.9780890424865. Accessed 1 Apr 2024. 10.1176/appi.ajp.23180001 36722117

[9] Nagata JM, Bojorquez-Ramirez P, Nguyen A, Ganson KT, Machen VI, Cattle CJ (2022). Sex differences in Refeeding among hospitalized adolescents and young adults with eating disorders. Int J Eat Disord.

[10] Strandjord SE, Sieke EH, Richmond M, Khadilkar A, Rome ES (2016). Medical stabilization of adolescents with nutritional insufficiency: a clinical care path. Eat Weight Disord.

[11] Whitelaw M, Nagata JM (2021). Nutritional considerations for boys and men with eating disorders. Eating disorders in boys and men.

[12] Garber AK, Cheng J, Accurso EC, Adams SH, Buckelew SM, Kapphahn CJ et al. Short-term outcomes of the study of refeeding to optimize Inpatient gains for patients with Anorexia Nervosa. JAMA Pediatr. 2021;175. 10.1001/jamapediatrics.2020.3359 PMC7573797 33074282

[13] Whitelaw M, Gilbertson H, Lam P-Y, Sawyer SM (2010). Does aggressive refeeding in hospitalized adolescents with anorexia nervosa result in increased hypophosphatemia?. J Adolesc Health.

[14] da Silva JSV, Seres DS, Sabino K, Adams SC, Berdahl GJ, Citty SW (2020). ASPEN Consensus recommendations for Refeeding Syndrome. Nutr Clin Pract.

[15] Friedli N, Odermatt J, Reber E, Schuetz P, Stanga Z (2020). Refeeding syndrome: update and clinical advice for prevention, diagnosis and treatment. Curr Opin Gastroenterol.

[16] Persaud-Sharma D, Saha S, Trippensee AW, StatPearls (2024). Refeeding syndrome.

[17] Reber E, Friedli N, Vasiloglou MF, Schuetz P, Stanga Z (2019). Management of Refeeding Syndrome in Medical inpatients. J Clin Med.

[18] Tresley J, Sheean PM (2008). Refeeding syndrome: recognition is the key to Prevention and Management. J Am Diet Assoc.

[19] Funayama M, Koreki A, Mimura Y, Takata T, Ogino S, Kurose S (2022). Restrictive type and infectious complications might predict nadir hematological values among individuals with anorexia nervosa during the refeeding period: a retrospective study. J Eat Disorders.

[20] Kells M, Gregas M, Wolfe BE, Garber AK, Kelly-Weeder S (2022). Factors associated with refeeding hypophosphatemia in adolescents and young adults hospitalized with anorexia nervosa. Nutr Clin Pract.

[21] Brown CA, Sabel AL, Gaudiani JL, Mehler PS (2015). Predictors of hypophosphatemia during refeeding of patients with severe anorexia nervosa. Int J Eat Disord.

[22] Sabel AL, Rosen E, Mehler PS (2014). Severe anorexia nervosa in males: clinical presentations and Medical Treatment. Eat Disord.

[23] Society for Adolescent Health and Medicine (2022). Medical management of restrictive eating disorders in adolescents and young adults. J Adolesc Health.

[24] O’Connor G, Nicholls D (2013). Refeeding hypophosphatemia in adolescents with anorexia nervosa: a systematic review. Nutr Clin Pract.

[25] Society for Adolescent Health and Medicine (2022). Refeeding Hypophosphatemia in Hospitalized adolescents with Anorexia Nervosa. J Adolesc Health.

[26] Garber AK, Cheng J, Accurso EC, Adams SH, Buckelew SM, Kapphahn CJ (2019). Weight loss and illness severity in adolescents with atypical Anorexia Nervosa. Pediatrics.

[27] Whitelaw M, Lee K, Gilbertson H, Sawyer S (2018). Predictors of complications in Anorexia Nervosa and atypical Anorexia Nervosa: degree of underweight or extent and recency of weight loss?. J Adolesc Health.

[28] Raj KS, Keane-Miller C, Golden NH (2012). Hypomagnesemia in adolescents with eating disorders hospitalized for medical instability. Nutr Clin Pract.

[29] Nagata JM, Vargas R, Sanders AE, Stuart E, Downey AE, Chaphekar AV (2024). Clinical characteristics of hospitalized male adolescents and young adults with atypical anorexia nervosa. Int J Eat Disord.

[30] Tipton K, Leas BF, Mull NK, Siddique SM, Greysen SR, Lane-Fall MB, et al. Interventions to decrease hospital length of Stay. Agency for Healthcare Research and Quality (AHRQ); 2021. 34644039

[31] Nagata JM, Golden NH, Peebles R, Long J, Murray SB, Leonard MB (2017). Assessment of sex differences in body composition among adolescents with anorexia nervosa. J Adolesc Health.

[32] Medical Management of Restrictive Eating (2022). Disorders in adolescents and young adults. J Adolesc Health.

