# Supplementary material for: Sex differences in electrolyte abnormalities indicating refeeding syndrome risk among hospitalized adolescents and young adults with eating disorders
Source: J Eat Disord. 2024 May 24;12:67. doi: 10.1186/s40337-024-01012-0 (PMC11127403; doi:10.1186/s40337-024-01012-0)
Supplement: Supplementary file 1 — Supplementary Material 1 [file 40337_2024_1012_MOESM1_ESM.docx]

| Appendix A. Electrolyte laboratory values of hospitalized adolescents and young adults with anorexia nervosa, restricting subtype, by sex | | | | | |  |
| --- | --- | --- | --- | --- | --- | --- |
| Characteristic | Total (N = 273) | Sex | |  |  | |
|  |  | Male (n = 30) | Female (n = 243) | p ^a^ | Effect size^b^ | |
| Admission serum electrolyte laboratory values, mean (SD) |  |  |  |  |  | |
| Magnesium | 2.2 ± 0.2 | 2.1 ± 0.2 | 2.2 ± 0.2 | .475 | 0.138 | |
| Phosphorus | 3.9 ± 0.6 | 4.0 ± 0.8 | 3.9 ± 0.6 | .218 | 0.239 | |
| Potassium | 3.9 ± 0.5 | 4.0 ± 0.4 | 3.9 ± 0.5 | .381 | 0.170 | |
| Refeeding electrolyte laboratory values, n (%) |  |  |  |  |  | |
| Refeeding hypomagnesemia (<1.8 mg/dL) | 55 (20.2) | 4 (13.3) | 51 (21.0) | .324 | 0.060 | |
| Refeeding hypophosphatemia (<3.0 mg/dL) | 25 (9.2) | 2 (6.7) | 23 (9.5) | .616 | 0.030 | |
| Refeeding hypokalemia (<3.5 mmol/L) | 34 (12.5) | 3 (10.0) | 31 (12.8) | .666 | 0.026 | |
| Combined refeeding syndrome risk*^c^* | 75 (27.5) | 6 (20.0) | 69 (28.4) | .331 | 0.059 | |
| Boldface indicates p < 0.05 |  |  |  |  |  | |
| *^a^* P-value is for t-tests for continuous variables or Pearson's chi square tests (or Fisher's exact test as appropriate) for categorical variables. | | | | | |  |
| *^b^* Cohen's d for continuous variables, Cramer's V for categorical variables. | | | | | |  |
| *^c^* A variable indicating refeeding hypomagnesemia or hypophosphatemia or hypokalemia | | | | | |  |
